# Indole-3-carbinol, a plant nutrient and AhR-Ligand precursor, supports oral tolerance against OVA and improves peanut allergy symptoms in mice

**DOI:** 10.1371/journal.pone.0180321

**Published:** 2017-06-30

**Authors:** Christiane Hammerschmidt-Kamper, Daniel Biljes, Katja Merches, Irina Steiner, Thomas Daldrup, Marianne Bol-Schoenmakers, Raymond H. H. Pieters, Charlotte Esser

**Affiliations:** 1 IUF – Leibniz Research Institute for Environmental Medicine, Düsseldorf, Germany; 2 Institute of Legal Medicine, Department of Forensic Toxicology, University Hospital of Düsseldorf, Düsseldorf, Germany; 3 Institute for Risk Assessment Sciences, Utrecht University, Utrecht, The Netherlands; Wayne State University, UNITED STATES

## Abstract

In general, dietary antigens are tolerated by the gut associated immune system. Impairment of this so-called oral tolerance is a serious health risk. We have previously shown that activation of the ligand-dependent transcription factor aryl hydrocarbon receptor (AhR) by the environmental pollutant 2,3,7,8-tetrachlorodibenzo-p-dioxin (TCDD) affects both oral tolerance and food allergy. In this study, we determine whether a common plant-derived, dietary AhR-ligand modulates oral tolerance as well. We therefore fed mice with indole-3-carbinole (I3C), an AhR ligand that is abundant in cruciferous plants. We show that several I3C metabolites were detectable in the serum after feeding, including the high-affinity ligand 3,3´-diindolylmethane (DIM). I3C feeding robustly induced the AhR-target gene CYP4501A1 in the intestine; I3C feeding also induced the *aldh1* gene, whose product catalyzes the formation of retinoic acid (RA), an inducer of regulatory T cells. We then measured parameters indicating oral tolerance and severity of peanut-induced food allergy. In contrast to the tolerance-breaking effect of TCDD, feeding mice with chow containing 2 g/kg I3C lowered the serum anti-ovalbumin IgG1 response in an experimental oral tolerance protocol. Moreover, I3C feeding attenuated symptoms of peanut allergy. In conclusion, the dietary compound I3C can positively influence a vital immune function of the gut.

## Introduction

Oral tolerance (OT) is a vital and unique feature mediated by the mucosal immune system. OT denotes that the immune system remains unresponsive to food antigens encountered in the gut. OT was first described many decades ago, and models of inducing OT in the mouse have shown that the antigen dose is important for the mechanism underlying OT. Thus, it was proposed that high doses of antigen in the diet result in anergic T cells, while low doses result in an induction of regulatory T cells (Treg) by dendritic cells (DC) of the small-intestine-draining mesenteric lymph nodes [1–3]. This rigid difference is challenged, however, by the finding that high numbers of Treg develop in high-dose tolerizing protocols [4], and the difficulties of providing direct proof of T cell deletion and anergy.

OT against food antigens is both local and systemic, and must be distinguished from local tolerance against gut residing commensal bacteria. Food allergies and celiac disease are thought to result from impaired oral tolerance induction. On the flip side, OT is a promising therapeutic approach against autoimmune reactions. These approaches aim at establishing OT against the disease-causing autoantigens by feeding the respective protein or peptide [5–7].

Retinoic acid (RA) produced by DC enhances the conversion of T cells into Tregs [8]. Furthermore, RA produced by DC imprints the a4ß7 integrin gut-homing surface markers on T cells [9]. RA is generated by retinal dehydrogenases, which are highly expressed in DC. Interestingly, the aryl hydrocarbon receptor (AhR), a ligand-activated transcription factor, can enhance RA-mediated differentiation in myeloblastic leukemia cells [10], and the physiological AhR ligand 6-formylindolo[3,2-b]carbazol (FICZ) enhances differentiation in these leukemia cells [11]. AhR-activation by the AhR ligand drug VAF347 shifts the monocyte-mediated differentiation of T cells to a T helper (Th)-22 phenotype, which may protect from autoimmunity [12, 13]. The AhR is an old evolutionary transcription factor. It is activated upon binding of a diverse range of small molecular weight chemicals, among them certain xenobiotics (such as planar polyhalogenated hydrocarbons), dietary compounds (flavonoids, glucosinolates) or endogenously generated tryptophan derivatives [14]. AhR signaling is critical for many immune functions. The AhR is highly expressed in gut epithelium and gut immune cells. The AhR is also involved in gut epithelial barrier integrity, differentiation of innate lymphoid cells, and the Th17/ Treg balance [15–17]. Of note, AhR-ligands are abundant in the diet, e.g., plant components such as flavonoids or glucosinolates, or in animal tissue in which xenobiotic ligands such as dioxins accumulate.

We had previously shown that exposure to 2,3,87,8-tetrachlorodibenzo-p-dioxin (TCDD) destabilized oral tolerance in mice. Thus, TCDD treated mice produced antibodies against the model antigen ovalbumin (OVA) after several boosting immunizations, despite previous tolerization [18]. TCDD is an environmental pollutant, and policies seek to reduce pollution and thereby exposure—including by dietary uptake—as much as possible. Certain plant-derived AhR-ligands are part of a healthy diet, however, and some are even sold as dietary supplements in an uncontrolled market. We therefore sought to investigate how oral dosing of one such AhR-ligand, indole-3-carbinol (I3C), affects oral tolerance, and whether this correlated with an increase in food allergies. We here report the surprising finding that the naturally occurring ligand I3C was able to boost oral tolerance against OVA and improved the anaphylactic score in a model of peanut allergy.

## Material and methods

### Ultra-high performance liquid chromatography coupled with photodiode array detection (UPLC-PDA)

I3C or its metabolites 3,3´-diindolomethane (DIM) and indolo[3,2-b]carbazol (ICZ) were quantified in blood serum samples up to 7 days by feeding with 2 g/kg I3C containing chow. Briefly, 50 μl serum was mixed with 500 μl tert-butylmethylether and 20 μl ISTD (4-methoxyindole; 0,5 ng/μl in MeOH) for organic extraction, followed by drying under a stream of nitrogen N_2_. The extracts were then split and dissolved in either 70% methanol (for I3C) or acetonitrile (for DIM/ICZ). Blank human serum samples were spiked with defined concentrations of all three analytes to obtain calibrators at levels of 25, 50, 100, 250, 500 and 1000 ng/ml serum. Analyses were performed on a Waters Acquity^®^ ultra high performance LC-PDA system using MassLynx^®^ software, and an Acquity UPLC^®^ HSS C18 1.8 μm; 2.1 x150 mm column. The detector type was an UPLC LG 500 nm. Sera from two mice were analyzed and quantified by linear regression of the peak-area ratios (analyte/internal standard).

### Mice and dietary intervention

We used C57BL/6 mice or conditional AhR^ΔCD11c^ and AhR^Δvillin^. We used littermates as WT controls. Mice were bred in our specific-pathogen free animal facility; mice were under a 12 hour/12 hour light–dark cycle, and had access to food and water *ad libitum*. I3C (2 g/kg) enriched normal chow (NC) was purchased from Sniff Special Diets (Soest, Germany). The 2 g/kg dose was chosen because it had been successfully used in other long-term dietary intervention studies of gut immune parameters dependent on AhR [16, 19]. The daily food uptake/per mouse was equal for both chows (data not shown), and the calculated daily I3C dose per mouse was approximately 5–6 mg.

To determine gene induction in the small intestine, a bolus of 200 μg or 20 mg I3C/mouse or 10 μg/kg TCDD, dissolved in DMSO and diluted in olive oil (10% DMSO and 90% Olive oil) was fed by gavage.

Peanut extract (PE) (30 mg/ml) was prepared from peanuts from the Golden Peanut Plant (provided by Intersnack Nederland BV, The Netherlands) as described previously [20]. Peanut extracts were checked for protein content by BCA analysis (Pierce, Rockford, IL). Cholera toxin (CT) was purchased from List Biological Laboratories, Inc. (Campbell, CA).

Mice were sacrificed by cervical dislocation for organ resections. All experiments were performed with the permission of the relevant governmental bodies in accordance with relevant German animal welfare laws (permission by LANUV, Az.:84–02.04.2012 A166).

### Oral tolerance induction

For tolerization, mice were gavaged with 20 mg of OVA (grade V;#A5503, Sigma-Aldrich, Munich, Germany) in PBS/20 g body weight on day 0 and day 2 of the experiment. Mice were then immunized i.p. day 9 and boosted on days 20, 27, 34, and 42 with 10 μgOVA/20 g body weight, dissolved in 50 μl of PBS and 50 μl of incomplete Freund´s adjuvant (IFA). Blood samples were taken on the days of immunization.

### OVA-ELISA

OVA-specific IgG1 antibodies in mouse serum were determined by ELISA. 96-well plates were coated with 100 μg/ml overnight at 4°C. Serum samples were titrated onto the plates and detected with goat anti-mouse IgG1 antiserum (Southern Biotech, Birmingham, USA) coupled to biotin. The ELISA was developed with avidin-HRP/TMB and values measured at 450 nm after the addition of 0.75 μl of H_2_SO_4_. All values are expressed related to a standard from pooled sera of mice immunized and boosted with OVA/IFA.

### Flow cytometry of lamina propria immune cells

To isolate immune cells from the Lamina propria, we adapted protocols available in the literature, leaving out a gradient step to avoid the possible selective loss of viable cells [21]. Briefly, the small intestine was resected, washed and cut into small pieces. The pieces were placed for 20 minutes in a solution containing 0.2 mg/ml collagenase and DNAse (Roche, Mannheim, Germany). The solution was vigorously vortexed and then filtered through a 100 μm strainer. Cells were washed in medium and counted in trypan blue. Fluorescence was measured on a FACSCanto-II^™^ flow cytometer (Becton-Dickinson, Heidelberg, Germany). At least 30,000 cells were collected in list mode. Debris, dead cells, and doublets were gated out by staining with Fixable Viability Dye eFluor 506 (eBioscience, Frankfurt/Main, Germany staining. Lymphocytes/leucocytes were gated out according to scatter characteristics. Finally, the frequency of DC was determined in the remaining population by MHCII (M5/114.15.2 BD-Biosciences, Heidelberg, Germany), CD11c (N418), and CD103 (2E7, eBioscience, Frankfurt, Germany)

### Peanut allergy

Allergic reaction to peanut extracts (PE) was performed as described [22, 23]. Briefly, mice were sensitized to PE by intragastric exposure to PE (6 mg PE, 200 μl/mouse) with CT (15 μg/mouse) on three consecutive days (day 0, 1, and 2) followed by weekly dosing (days 7, 14, 21, and 28). One day before termination, mice were challenged i.p. with PE (10 μg PE/g body weight). Control mice received PBS only during sensitization and PE for challenge. Mice were fed chow containing I3C 2 g/kg throughout the experiment. Mice were scored for allergic symptoms on a 1–4 scale (1 = scratching at the snout, 2 = decreased activity, some swelling around nose, 3 = longer than 1 minute of no motions, 4 = no reaction upon touching of fibrillae, 5 = tremor and death—animals were killed before reaching this stage). The mice’s rectal temperatures were measured at the time points indicated. Blood samples for measuring PE-specific IgG and IgE were taken three days after challenge.

### RT-PCR of RNA from small intestine

Small intestine pieces were stored at -80°C. Tissue was homogenized in RNAmagic (BioBudget, Krefeld, Germany) with one 3 mm stainless steel or tungsten carbide bead (Qiagen, Hilden, Germany) at 30 Hz for 1 min using the TissueLyserII (Qiagen, Hilden, Germany). RNA was extracted from the homogenate by chloroform/isopropanol, after which the pellet was washed in ethanol and finally resuspended in water. RNA was reverse transcribed with MMLV reverse transcriptase. Quantitative PCR reactions were performed with the Rotor-Gene SYBR Green PCR Kit (Qiagen, Hilden, Germany) in a Rotor-GeneQ thermo cycler (Qiagen, Hilden, Germany). The following primers were used: Cyp1a1-For 5’-TCCTTGCATGTCCATGTTTC-3’, Cyp1a1-Rev 5’-TGCATAAGCAAAATACAGTCCA-3’, RPS6-For 5’-TACTGTGCCTCGTCGGTTG-3’, RPS6-Rev 5’-TGAATCTTGGGTGCTTTGGT-3’, AhR-For 5’-AGACCGGCTGAACACAGAGT-3’, AhR-Rev 5’-GTCAGCAGGGGTGGACTTTA-3’, Aldh1a1-For 5’-TGTGGGAATACCGTGGTTGTC-3’, Aldh1a1-Rev 5’-GTGAAGAGCCGTGAGAGGAG-3’ (Vicente-Suarez et al., 2015), Aldh1a3-For 5’-GAGCAGCAATTTCCTCCCATC-3’, Aldh1a3 5’-GAGCCGGTGAAGGCTATCT-3’ (Vicente-Suarez et al., 2015), GAPDH-For 5’-CGTCCCGTAGACAAAATGGT-3’, GAPDH-Rev 5’-TTGATGGCAACAATCTCCAC-3’. Expression levels were calibrated to the expression of *rp*s6 [24] in the same sample using the 2^ΔΔC^_T_ method [25].

### Statistical methods

Data were analyzed with GraphPad Prism^™^ using student’s t-test or ANOVA followed by Tukey’s or Sidak’s multiple comparison test as indicated in the results section. Levels of significance are indicated as * = P≤0.05, ** = P≤0.01, *** = P≤0.001, **** = P≤0.0001

## Results

### Oral exposure to I3C induces AhR-target gene CYP1A1 in the small intestine

**Fig 1:**
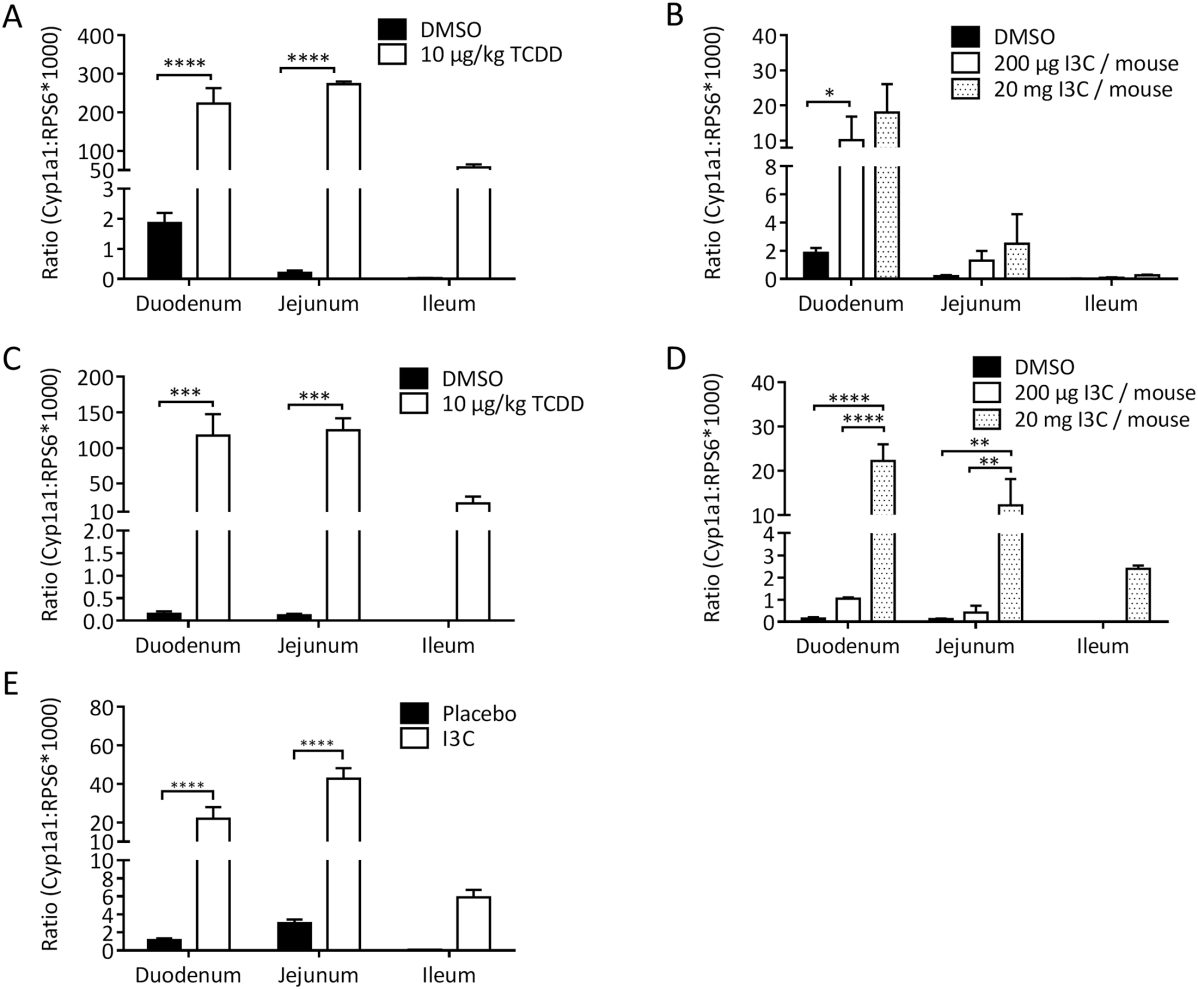
CYP induction in small intestine. A-D: Mice were fed by gavage with TCDD, I3C or DMSO (as solvent for TCDD) at the indicated doses. Shown is *cyp1a1* expression relative to the housekeeping gene *rps6* in the three sections of the small intestine 4 hours after gavage (A, B), or 24 hours after gavage (C, D). Note the scale differences for TCDD and I3C. N = 3; Mean ± SEM. Two-way ANOVA followed by Sidak’s multiple comparison test. *≤0.05, ** ≤0.01, *** ≤0.001, **** ≤0.0001. E: Mice were fed for 49 days with chow containing 2 g/kg I3C. On day 49, RNA was isolated from small intestine and *cyp1a1* expression was analyzed by quantitative PCR. The results of two pooled independent experiments are shown. N = 11–15. Mean ± SEM. Two-way ANOVA followed by Sidak’s multiple comparison test. *≤0.05, ** ≤0.01, *** ≤0.001, **** ≤0.0001.

We first assessed whether feeding mice with I3C induces the AhR-target gene *cyp1a1* in the small intestine. We gavaged mice with 200 μg or 20 mg I3C per mouse; we then sacrificed the mice 4 or 24 hours later. Control mice were fed solvent alone (low control) or 10 μg/kg body weight TCDD (high control). As shown in Fig 1, the lower dose of 200 μg of significantly induced *cyp1A1* gene expression in the duodenum after 4 hours. In the more distal small intestine sections, induction was visible, but did not reach significance. In contrast, the high 20 mg I3C dose induced strong *cyp1a1* expression along the entire small intestine (in duodenum, jejunum and ileum) at both 4 hours and 24 hours. Except for the ileum, the increase was significant. The I3C effect was long-lasting and still detectable 24 hours after gavage. The *cyp1a1* induction, however, was an order of magnitude lower than the induction observed by 10 μg of TCDD. Oral tolerance protocols take several weeks. I3C is metabolized and thus a single bolus may not be enough for continuous AhR-activation. We therefore tested whether the enrichment of mouse chow with 2 g/kg (resulting in an approximate dose of 5–6 mg/mouse/day) induces AhR-target gene *cyp1a1*. Mice were fed for 49 days with this chow, and as shown in Fig 1E, *cyp1a1* induction was high along the entire small intestine, significantly in the duodenum and jejunum. Thus, continuous feeding of an AhR ligand does not lead to exhaustion, but to persistent AhR-activation in the gut. We note, however, that the single bolus of 20 mg I3C/mouse and the long-term dietary intervention led to approximately the same AhR induction over the control, indicating a plateau level of AhR-activation (compare Fig 1D with 1E). The expression of the *AhR* gene itself was not affected by I3C feeding (data not shown). We used the dose of 2 g/kg chow in further dietary intervention studies.

### Metabolites of dietary I3C are detectable the serum

**Fig 2:**
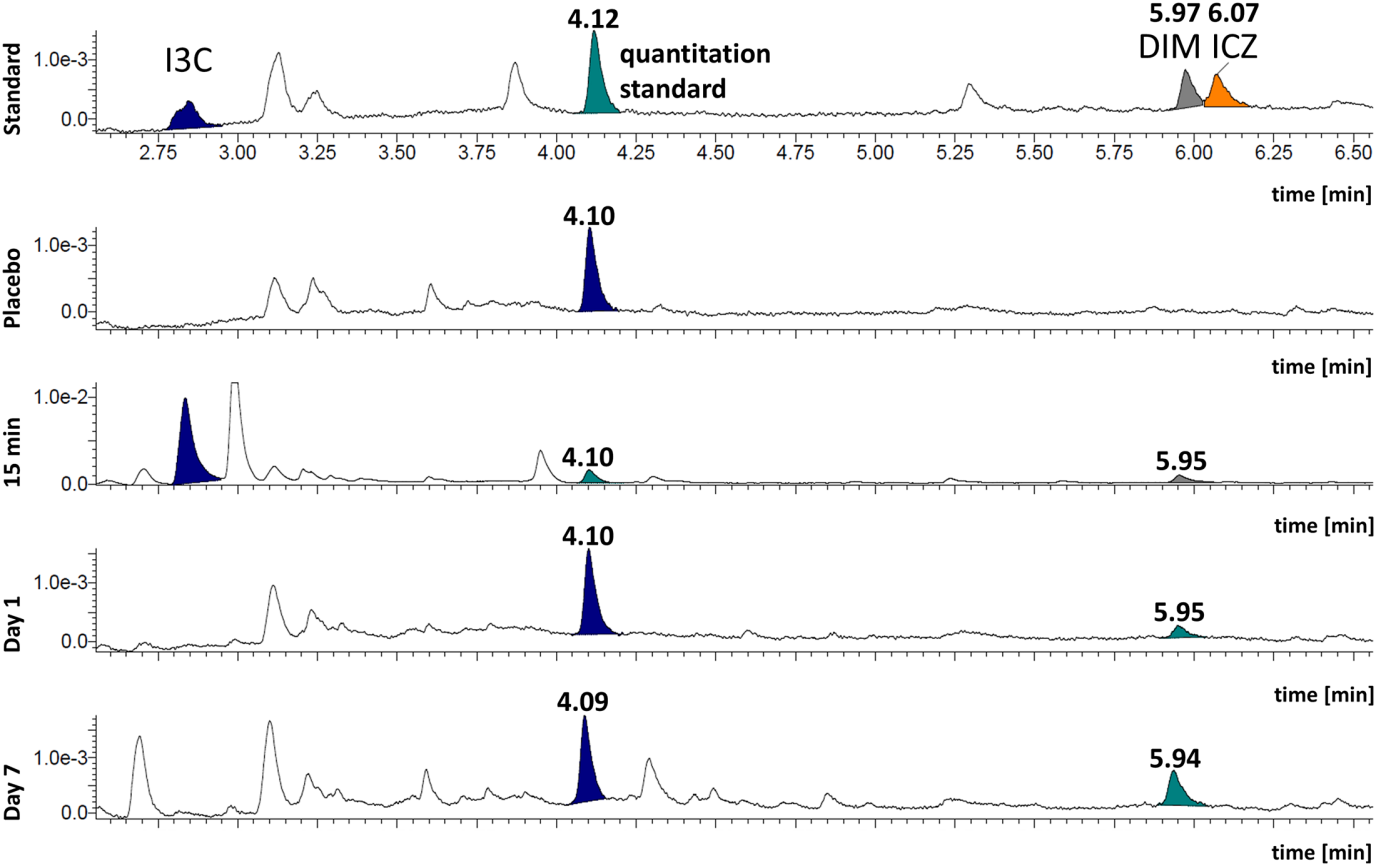
Quantification of I3C and metabolites in mouse serum after oral uptake. Mice were fed for up to 7 days with chow containing 2 g/kg I3C. For the 15 minute value, mice received 5 mg/I3C per mouse diluted in 10%DMSO/olive oil by gavage. Serum samples were prepared and analyzed for the presence of I3C, DIM, and ICZ. Sera were spiked with 4-methoxyindole as the internal standard to calibrate for possible differences in extraction efficiency. Note that I3C is detectable only after the bolus feeding, but not when I3C was given in the chow, and is thus eaten in smaller amounts over the entire day. Y-axis: arbitrary units.

We took serum samples 15 minutes, 2 days and 7 days after giving I3C by gavage (15 min) or via supplemented chow; we analyzed the samples by UPLC coupled to UV-detection. I3C is known to convert in vitro within minutes in an acid solution into a complex mixture, with approximately 2–6% DIM, and <0.01% ICZ [26]. Similarly, the group of Bjeldanes reported that DIM and ICZ were present in gut tissue after I3C gavage (yields of 0.1% and <0.01%, respectively). Confirming and extending their results, we analyzed the serum and found that the I3C metabolite DIM was easily detectable, but ICZ was below our limit of detection (Fig 2). In addition to DIM, eight possible metabolites of I3C were detected; however, we did not characterize them further. The concentrations of I3C, DIM and metabolites detected at the end of the feeding periods are shown in Table 1.

**Table 1:** Quantification of I3C metabolites in serum.

	concentration [ng/ml]^a^									
	I3C	DIM	Met1 2.49 min^b^	Met2 3.28 min	Met3 3.87 min	Met4 4.23 min	Met5 4.39 min	Met 6 4.52 min	Met 7 4.78 min	Met 8 5.16 min
15 min^c^	~ 2000	182	178	-	1450	158	-	-	-	132
d1^c^	-	56.5 ± 1.5	-	184.5 ± 27.5	-	-	27 ± 13	22 ± 8	27 ± 4	-
d7^c^	-	80 ± 42	-	84.5 ± 10.5	-	-	123.5 ± 81.5	17 ± 12	71 ± 30	-

^a^ Linear calibration curves were established for serum samples at concentrations from 25 to 1000 ng/ml. The equation was y = 0.0033x + 0.0742 (R^2^ = 0.9976) for I3C, y = 0.0067 x 0,1422 (R2 = 0,9894) for DIM and y = 0,0126 x –0,4738 (R2 = 0,9788) for ICZ. From this the serum concentration were calculated as [ng/ml] for peaks at the shown retention times (in minutes). Several unidentified metabolites (Met 1- Met 8) are shown as well.

^b^ Time in minutes when a peak became detectable, a measure for a distinct substance.

^c^ The 15 min value derives from one mouse gavaged with a bolus of 5 mg/mouse I3C; mice were sacrificed 15 min later. The values for d1 and d7 are from mice fed via their chow, which contained 2 g/kg, and thus led to an approximate dose of 5–6 mg/mouse/per day. N = 2 mice, mean values ± SD are shown.

### Dietary intervention with I3C licenses oral tolerance against OVA

**Fig 3:**
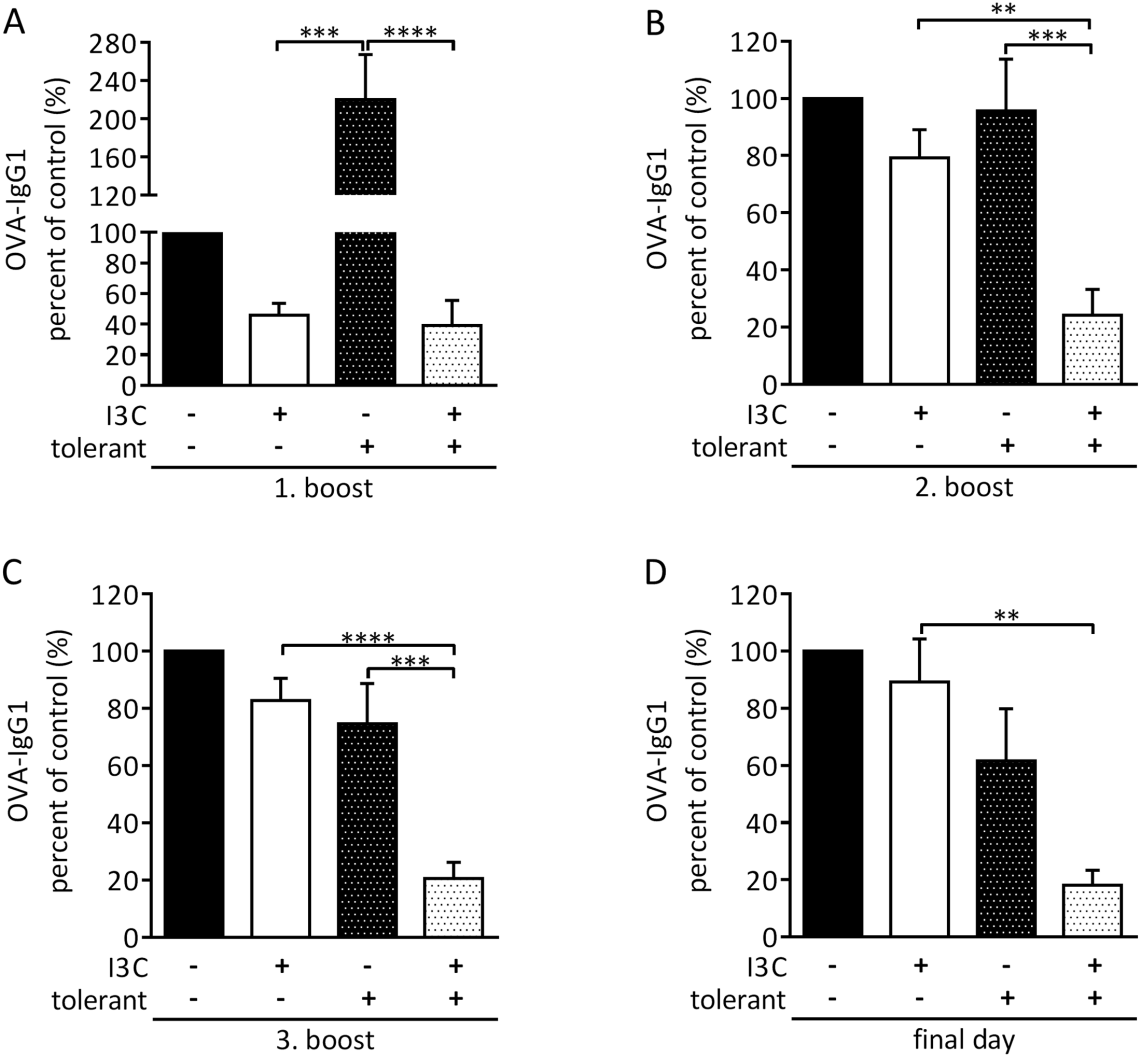
I3C in the diet licenses oral tolerance. Mice were fed a placebo (black bars) or I3C containing diet (white bars) and were either not tolerized (PBS = control) or tolerized with OVA (dotted bars). Mice were immunized and then boosted 2 (A), 3 (B), and 4 weeks (C) later. Antibody titers were determined at the day of each boost and one week after the third boost (D). Antibody titers are depicted as % of placebo/PBS-treated animals that were set to 100%. Data are pooled from three independent experiments. N = 12–13 mice/group. Mean ± SEM. One-way ANOVA followed by Tukey’s multiple comparison test. *≤0.05, ** ≤0.01, *** ≤0.001, **** ≤0.0001.

Several naturally occurring ligands of AhR, including indoles, reportedly ameliorated autoimmune diseases such as EAE or allergic asthma [27, 28]. We developed an OVA feeding protocol in such a way that the OVA dose was no longer high enough to induce oral tolerance in mice fed normal chow. Mice on normal or I3C enriched chow (starting already at day minus 7) were fed 20 mg OVA/20 g body weight on days 0 and 2, followed by immunization and 3 boosts. Fig 3 shows that in this situation, feeding of I3C led to five times lower titers of anti-OVA IgG1 antibodies compared to normal chow-fed control mice, i.e., I3C enrichment of the chow supported the formation of oral tolerance.

### Characterization of gut immune cells upon I3C feeding

**Fig 4:**
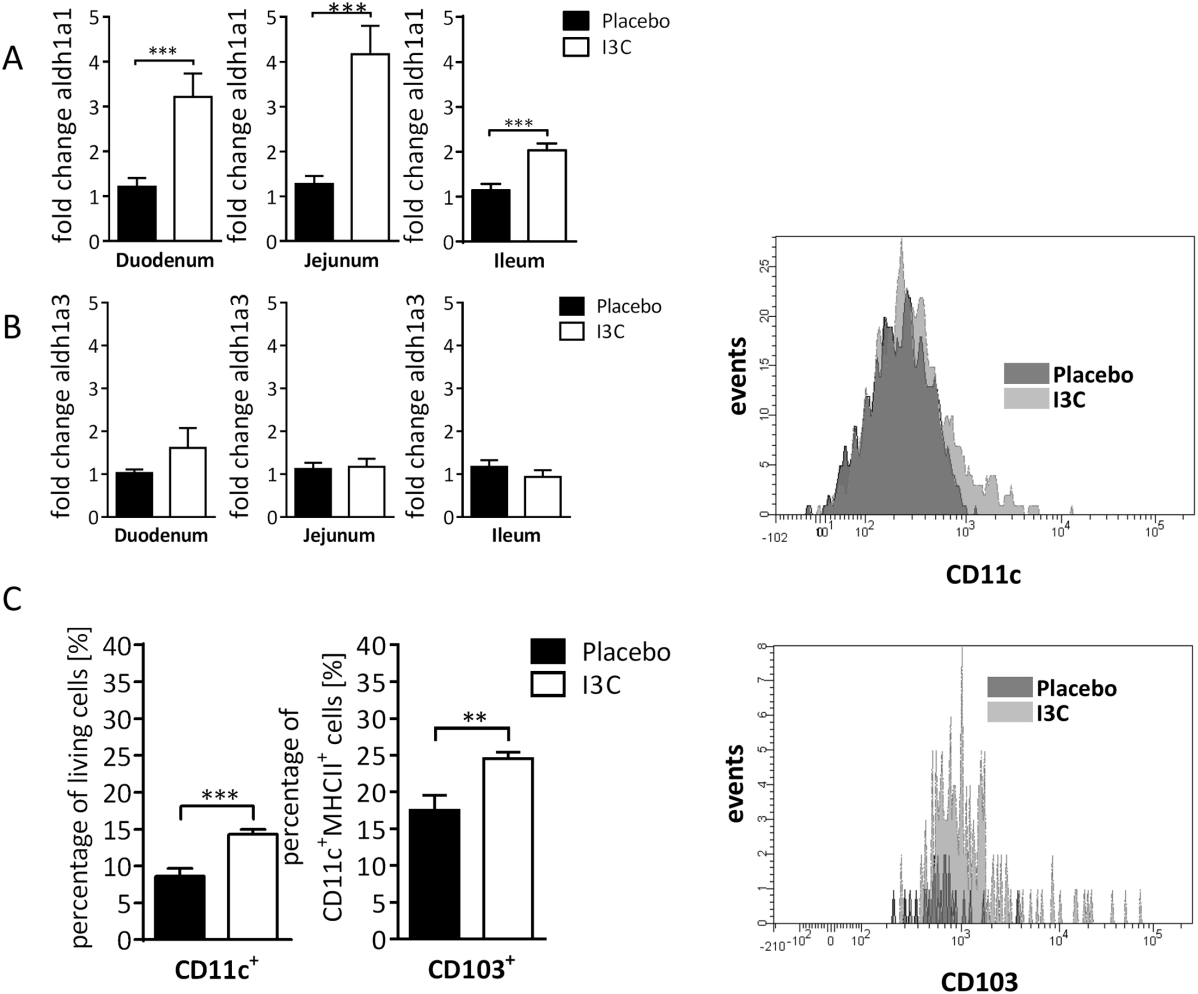
Increase of DC in lamina propria and increased expression of *aldh* in the small intestine. A-B: *aldh1* expression after continuous feeding of I3C. Mice were fed for 49 days with normal or I3C enriched chow and expression of retinaldehyde dehydrogenase *aldh1a1* (A) and *aldh1a3* (B), analyzed by quantitative PCR. Fold change compared to housekeeping gene GAPDH is shown. Shown is the mean ± SEM of pooled data from two independent experiments. C-D: mice were fed continuously for 28 days normal chow (“placebo”) or I3C-enriched chow before tolerance induction. Samples were taken on day 21, i.e., 49 days after start of dietary intervention. For gating strategy see Material and methods C: Percentage of CD11c^+^ (MHCII positive or negative) in living lamina propria cells of the small intestine (left), and the CD103+ subpopulation in CD11c+MHCII (right) DC in the lamina propria. D. typical histograms N = 9. Mean ± SEM. Unpaired Student’s t-test. *≤0.05, ** ≤0.01, *** ≤0.001.

**Fig 5:**
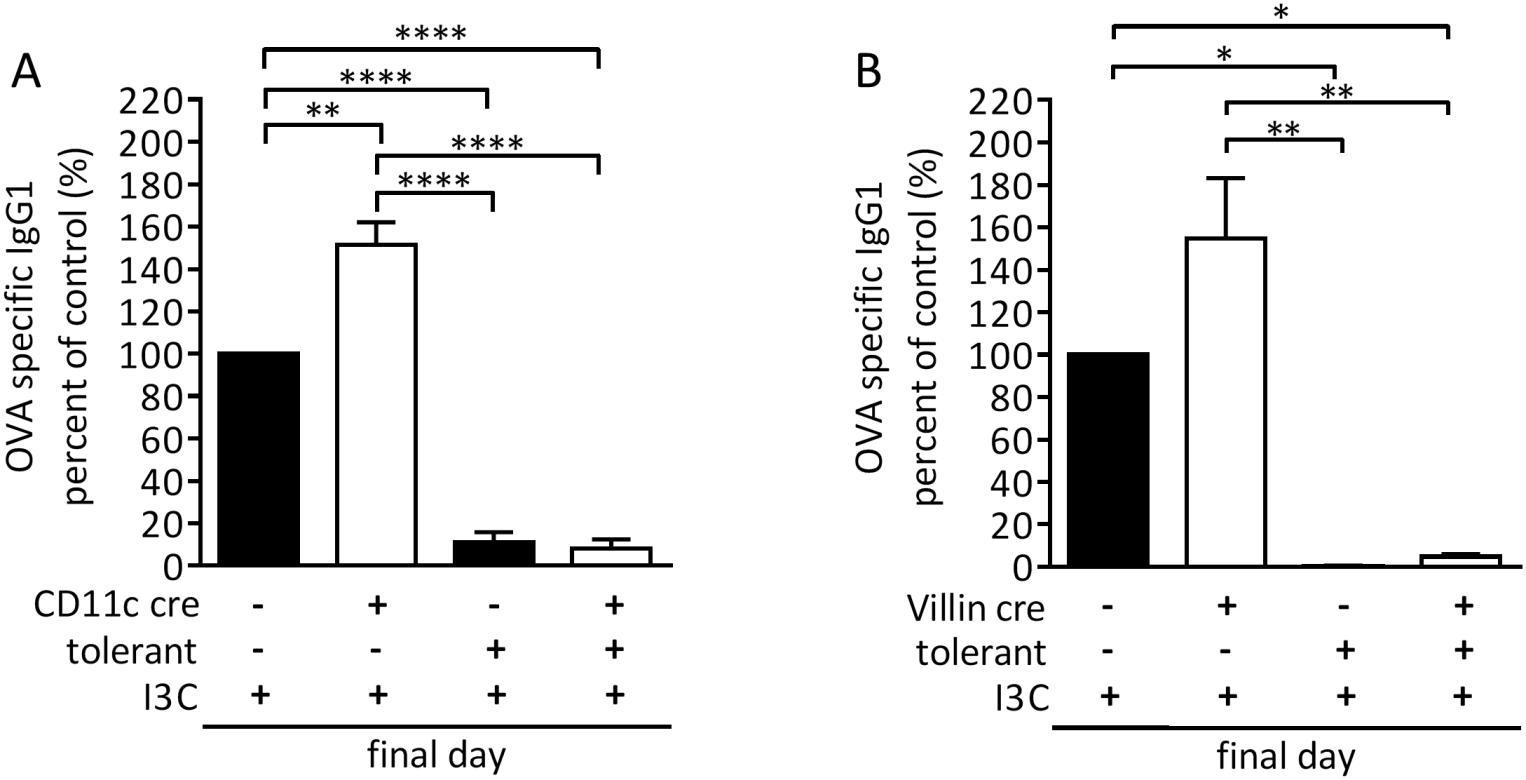
OT in conditional AhR-deficient mice. OVA specific IgG1 antibody titers of AhR^ΔCD11c^ (A) and AhR^Δvillin^ (B) mice that lack the AhR in dendritic cells or intestinal epithelial cells, respectively. AhR^ΔCD11c^ and AhR^Δvillin^ mice (= cre^+^; white bars) and WT littermates (= cre^-^; black bars) were fed an I3C containing diet and either not tolerized (PBS = control) or tolerized with OVA. Antibody titers were determined at the end of the experiment. Antibody titers are depicted as % of I3C/cre^-^/PBS-treated animals that were used as controls. Mean ± SEM. One-way ANOVA followed by Tukey’s multiple comparison test. *≤0,05, ** ≤0,01, *** ≤0,001, **** ≤0,0001.

The lamina propria is the mucosal effector site where many DC reside and contribute to gut homeostasis and a non-inflammatory state [29]. DCs are MHC-II^+^CD11c^+^CD103^+^, but several subsets—possibly differing in functions—exist and can be identified by further surface molecules. We analyzed lamina propria dendritic cells by flow cytometry in I3C fed mice versus control mice. Mice were fed for 28 days with 2 g/kg I3C supplemented chow and during the following 21-day tolerizing protocol (see Material and methods). Interestingly, the percentage of CD11c+ increased significantly in the lamina propria in these mice fed I3C. Within this subset, the number of CD103+MHC-II+, i.e., those DC with tolerogenic properties, increased compared to control DC (Fig 4A and 4B). At the same time, feeding of I3C induced retinaldehyde dehydrogenase *aldh1a1* in the small intestine Fig 4C. In contrast, a*ldh1a3* was not induced by I3C feeding (Fig 4D). The enzyme Aldh1a1 converts vitamin A to retinoic acid, which in turn, induces TGFβ production by DC, and thus is critical for the generation of regulatory T cells in the gut and to gut-homing of Treg [30]. AhR activity can drive Aldh1 [31], although the evidence is indirect so far. Aldh1 enzymes are expressed in both intestinal epithelial cells and in DC [32–34]. We therefore tested “I3C tolerogenicity” in conditional AhR-deficient mice, where AhR is lost either in intestinal epithelial cells (AhR^ΔVillin^) or in CD11c (AhR^ΔCD11c^). The results show that in both mouse lines, I3C supported tolerance, indicating that the presence of AhR in either cell type alone is not a sine qua non for the positive I3C effect (Fig 5). AHR-null mice were not tested upon I3C feeding as their digestion and detoxification would not be comparable due to their patent ductus venosus [35, 36]

### Peanut allergy after I3C feeding

**Fig 6:**
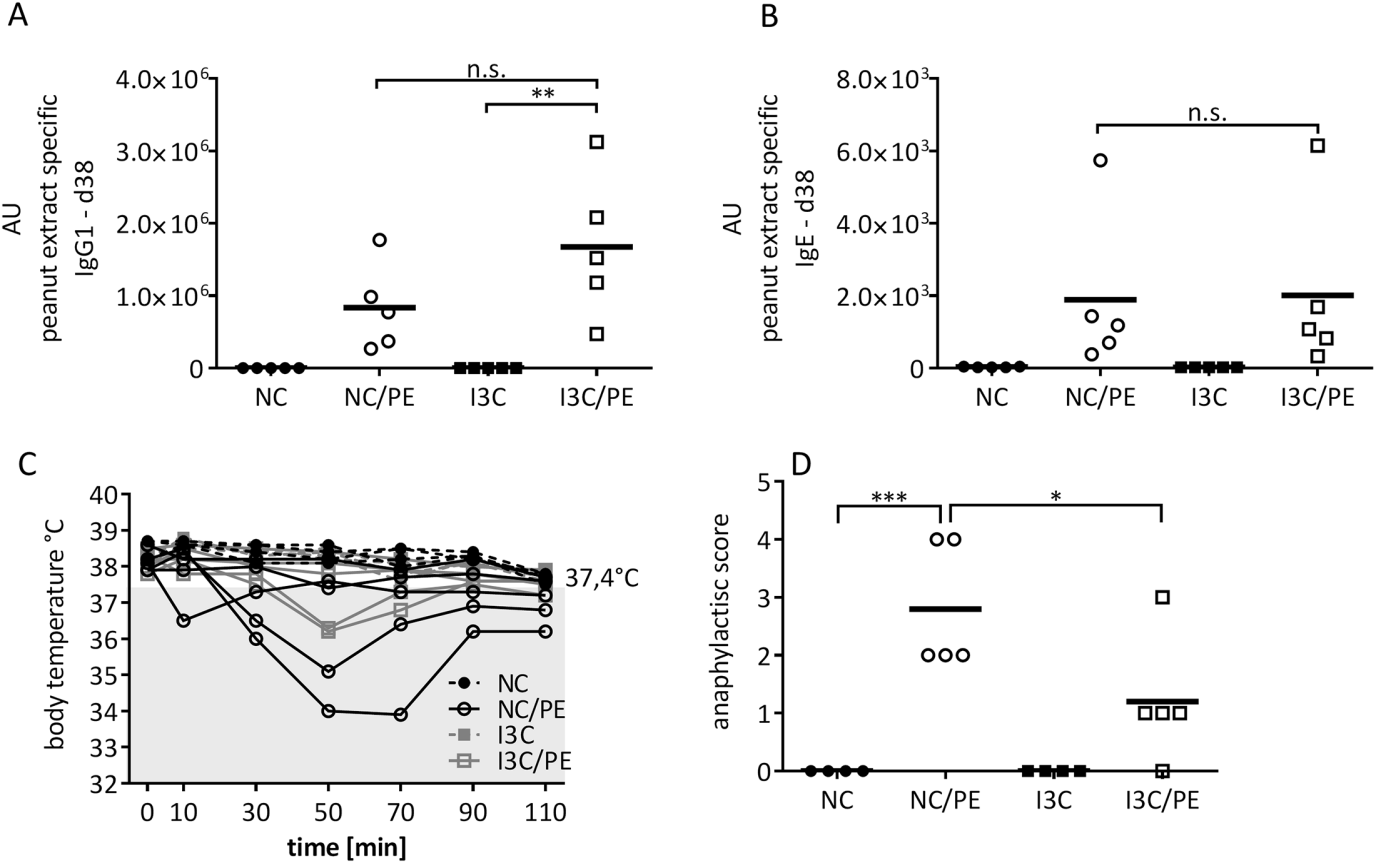
I3C feeding dampens parameters of peanut allergy in mice. Mice were fed with normal (NC) I3C-enriched chow (I3C), sensitized to peanut extract, and then challenged by an i.p. injection with peanut extract (PE). A: IgG1 B: IgE antibody titers. C: rectal temperatures were taken after peanut injection. Mean rectal body temperature for placebo control was 38.3°C. The number of mice with a drop in temperature of >2% (considered a symptom of allergy), i.e., with a temperature below 37.4°C, were counted at 10, 30, 50, 70, 90, and 110 minutes. D: Anaphylactic scores. 0 = no symptoms; (N = 4 for control groups, N = 5 for PE groups. Mean± SEM is shown; A, B, C. statistical significance calculated with One-way ANOVA followed by Tukey’s multiple comparisons test: *≤0,05, **≤0,01, ***≤0,001,. NC = normal chow; I3C = 2 g/kg I3C supplemented chow graph.

TCDD exposure suppresses the allergic reaction in a model of peanut allergy in mice [37]. We therefore tested this model in I3C fed mice. Mice were first sensitized and then challenged i.p. with peanut extract; we then measured anti-peanut IgG1 and IgE antibodies in the serum. In contrast to the TCDD exposure [37], feeding I3C did not suppress peanut allergy (Fig 6). Peanut extract induced a strong antibody response for both isotypes, but feeding of I3C had no effect on the response strength (Fig 6A and 6B). The strength of an allergic response can be assessed by the typical sudden drop in body temperature upon antigen challenge, and by scoring the bodily reaction (from 1–5, see Material and methods). The mean body temperature of the mice at the onset of the challenge was 38.23 ±0.26°C. The drop in body temperature is shown in Fig 6C. Indeed, only 2/5 mice fed with I3C but 4/5 mice on normal chow had a drop in body temperature of more than 2% (i.e., ≤37.4°C) upon allergen challenge. Additionally, when scoring the overall body reaction, a significant improvement by I3C was observed. While the allergic reaction in untreated mice resulted on average in a score of 3, i.e., swelling around the nose and >1 min motionlessness, the I3C-fed mice scored 1, i.e., they displayed only some scratching (Fig 6D).

Thus, our data in the two models of gut-mediated immune responses indicate that I3C can improve both oral tolerance and ameliorate the symptoms of a food allergy. Together, this suggests that dietary AhR-ligands are part of gut immune homeostasis.

## Discussion

The health benefits of plants of the *Brassica* family are well known. The focus in the respective research is mainly on the anti-tumorigenic properties, however, where studies demonstrated beneficial effects of broccoli on some (but not all) cancers [38]. The phytochemicals mediating these effects are often glucosinolates, indoles, and their various derivatives, in particular their acid-derivatives. Indoles can bind to the AhR, leading to gene expression, e.g., of phase I and phase II enzymes [39], and eventually cellular and systemic responses. In the rat, increased CYP1A1 enzyme activity has been shown in the liver after feeding of 57 mg I3C/kg for 7 days [40]. We extended this study to show that oral exposure leads to induction of the *cyp1a1* along the murine small intestine, i.e., our site of immunological interest. Moreover, we found that induction did not subside upon long-term exposure of a comparatively moderate dose, indicating constantly high AhR-activity by the diet. I3C is converted to several metabolites in the acid environment of the stomach, most abundantly into DIM, as well as into other products [26, 41]. Both I3C and DIM are AhR ligands, with binding affinities of approximately 2x10^-10^ (ICZ) and 9x10^-8^ (DIM) [26, 41], compared to 10–12 M for TCDD. Of note, we detected I3C derivatives in serum, i.e., they can transfer systemically. This extends older studies, which analyzed I3C derivatives DIM and ICZ locally in the gut and feces within a day of I3C feeding [26].

In contrast to cancer, very few studies examined immunological consequences of dietary AhR-ligands. AhR is important for the immune system and critically contributes to the balance of regulatory T cells and Th17 pro-inflammatory T cells [17, 42, 43]. Indeed, it was shown that removal of AhR ligands from the diet mimics the adverse effects of AhR-deficiency in the gut [15, 16], such as failure to expand innate lymphoid cells after birth. Re-addition was able to rescue the effect in wild-type, but not in AhR-deficient mice, demonstrating its AhR-dependency [16]. We here investigated the effects of I3C on the vital immune function of oral tolerance, and found that dietary exposure of I3C licensed oral tolerance under conditions that would not normally induce this immunosuppressive state. Thus, I3C exposure had the opposite outcome of TCDD exposure, which broke tolerance. Rather, the I3C effect we observed is reminiscent of an experiment by the group of Howard Weiner, who applied 2-(1'H-indole-3'-carbonyl)-thiazole-4-carboxylic acid methyl ester (ITE) and the autogenic peptide MOG_35-55_ embedded in nanoparticles to mice. This protocol of oral tolerization treatment improved the outcome of experimental autoimmune encephalitis (EAE), by inducing Treg [44]. Supporting tolerization or induction of tolerance is of great interest as a therapeutic approach [2, 45]. Beyond oral tolerance, it has also been tested in other tolerizing systems, e.g., successfully using anterior-associated immune deviation to improve collagen-induced arthritis or EAE [46, 47]. Amelioration of EAE by injections of AhR ligands have been studied for several years [48, 49]; the discussed mechanism includes both the induction of Treg via changes in DC tolerogenicity, or the induction of T cell cycle arrest by certain micro RNAs [49]. As a caveat, the group of Nagarkatti showed that dietary I3C and DIM suppressed a DTH-response against a model protein, while the high-affinity ligand FICZ did not [28].

Why did I3C behave so differently from TCDD in our experimental OT model and why do differences exist between AhR-ligands in the other studies? For OT, several possibilities exist, namely (i) the antigen dose, (ii) exposure timing, or (iii) nature of the AhR-binding peculiars. To start with the latter, the differing effects of ligands on AhR-activation outcome are well-known but little understood, and still highly controversial [50]. Whether different ligands bind to AhR at slightly differing sites and with differing outcomes on its ligand-induced conformation changes, DNA-binding capacity or resistance to down-stream degradation is not known, and must remain speculative as only the PAS-A domain of AhR has been crystallized so far [51–53]. Another obvious explanation is the persistence of TCDD in the body, while other ligands are often quickly degraded. By feeding I3C continuously (which continuously triggered high cyp1a1 expression), we tried to equalize this parameter. Indeed, our data show that continuous feeding of I3C results in a plateau level of DIM in the serum. Thus, although DIM has an approximately three orders of magnitude lower binding affinity than TCDD, the dose is effective. Recently, we have found that the skin barrier can also be improved by I3C feeding; thus, I3C derivatives also can act far beyond the gut itself (Haas et al, in press).

Oral tolerance is foremost an observable phenomenon: food contains numerous proteins (>100 g protein per day in an average human diet) that can serve as antigens and are immunogenic if encountered under immunizing conditions; yet, we normally do not react to food antigens [54, 55]. Models of experimental oral tolerance (EOT) in animals suggested that the dose and feeding regimes can influence which mechanism is triggered, i.e., either the induction of regulatory T cells or the induction of T cell anergy [1, 2, 56]. Possibly, both mechanisms occur side-by-side in a real-life situation, and possibly there is also a “no observed effect level” dose, i.e., where food antigens might be too rare to necessitate an active oral tolerance response. In our hands, comparatively small changes in the antigen exposure scheme resulted in a situation in which tolerance was suboptimal but could be unmasked by I3C. Both the humoral and cellular immune responses are triggered in EOT. It involves “stepwise and anatomically defined processes of induction, maintenance and migration of Tregs” [57]. Central to the process are CD103^+^DC of the gut lamina propria, which take up antigen, transport it to MLN and induce a gut homing phenotype in T cells. The cytokine milieu can influence DC tolerogenicity depending on the local availability and context. For instance, GM-CSF can have both pro- and anti-inflammatory properties [58]. Lack of GM-CSF, which in the gut is mainly produced by RORγt(+) innate lymphoid cells (which, in turn, may need AHR for their development [16]), can lead to low numbers of Treg cells and impaired oral tolerance [59]. CD103^+^ DC derived RA supports the conversion of naïve T cells into regulatory T cells and helps to maintain their phenotype [29, 60, 61]. In a very recent study, the group of Joshua Mezrich demonstrated that mice fed an I3C-enriched diet had more Tregs, ILC3s and γδ T cells in cecal cells of the gut, associated with a better protection from *Clostridium difficile* infection [35]. We note, however, that in an in-vitro study where CD103+DC were differentiated from bone marrow cells, the addition of DIM suppressed their ability to induce Treg [62]. This result provides additional evidence of the importance of tissue spatial context and dose. While we failed to see a significant increase in Treg in the intestinal tissue in our study (data not shown), we identified increased frequencies of CD11c+ and CD103+ cells by I3C in our OT model. Moreover, the key enzyme for RA production was significantly higher in all sections of the small intestine after continuous exposure to I3C. Together, our findings offer an explanation of why oral tolerance was induced upon long-term I3C feeding, and can be tested further. Of note, epithelial cells are also an important source of RA and promote CD103+ DC differentiation [63]. Our results with I3C-fed conditional AhR-deficient mice (DC or IEC specific) support the argument that AhR-deficiency in only one of these cell types is not sufficient to loose I3C-enhanced tolerance.

Finally, we tested the effects of dietary I3C on peanut allergy. Food allergies can be understood as the breakdown of oral tolerance, resulting in systemic anaphylaxis and usually a Type I allergic response. Food allergies result in symptoms ranging from mild itching, to abdominal pain, bronchospasms and even anaphylactic shock. While difficult to estimate, it is thought that food allergies affect approximately 2–5% of the European population [64]. We found that feeding of I3C ameliorated the allergic symptoms in our mice, but not the sensitization (i.e., antibody formation). Thus, there seems to be no (protective) effect of I3C regarding allergy induction and B cell activity, which is in line with results regarding peanut allergy in AhR-KO mice [37]. Conceivably, challenge responses are more easily influenced by interventions compared to the antibody levels, i.e., sensitization itself. This has also been observed in other types of intervention [65], although we do not yet have a mechanistic explanation for this phenomenon. Possibly, I3C or its metabolites directly affect mast-cells, or interfere with mast-cell/Treg interaction.

While we only tested one dose regime, it is possible that more I3C could have even higher positive effects. Thus, more research is needed regarding other doses and timing. Importantly, while indoles have positive health effects and have even been clinically tested in certain settings as safe [66], they can turn cancerogenic at too high doses [38].

In conclusion, we showed that dietary I3C or its metabolites such as DIM activate AhR in the gut and contribute to the generation of anti-inflammatory milieu in the gut. This supports vital immune functions of oral tolerance and may ameliorate or prevent food allergies. We thus provide a new angle on the positive health effects of this important plant compound.

## Abbreviations

AhR: aryl hydrocarbon receptor
DC: dendritic cell
DIM: 3, 3'-diindolylmethane
FICZ: 6-formylindolo[3,2-b]carbazol
I3C: indolo-3-carbinol
ICZ: indolo[3,2-b]carbazol
OT: oral tolerance
OVA: ovalbumin
PE: peanut extract
RA: retinoic acid
SC: Standard chow
TCDD: 2,3,7,8-tetrachlorodibenzo-p-dioxin
